# The clinical implications of ear canal debris in hearing aid users

**DOI:** 10.12669/pjms.303.4742

**Published:** 2014

**Authors:** Foster Tochukwu Orji, Emmanuel O. Onyero, Christian Ejiofor Agbo

**Affiliations:** 1 Foster Tochukwu Orji, MBBS, FWACS, FMCORL, Department of Otolaryngology, University of Nigeria Teaching Hospital Enugu, Nigeria.Department of ENT, Federal Medical Center Umuahia, Nigeria.; 2 Emmanuel O. Onyero, MBBS, Department of ENT, Federal Medical Center Umuahia, Nigeria.; 3 Christian Ejiofor Agbo, BMLS, MSc Microbiology, Department of Laboratory Services, Federal Medical Center Umuahia, Nigeria.

**Keywords:** Hearing aid, Ear canal debris, Ear irritation, Microbial flora

## Abstract

***Objective*** **:** The ear irritations suffered by hearing aid (HA) users are yet to be related to the clinical state of canal. We undertook this study to examine the nature of debris and the microbial flora of ears of hearing aid users, as well as evaluate the determinant factors of ear irritation in this population.

***Methods*** **:** An observational clinical study was carried out involving 32 unilateral hearing aid users recruited from ENT clinic of a tertiary referral center. Each subject underwent otoscopic assessment of canal debris and microbial analysis of swab cultures taken from the hearing aid-wearing ear and contralateral normal ear without hearing aid.

***Results*** **:** Canal debris [wax (28%), fungal deposits (19%), bacteria exudates (13%)]. as well as microorganisms were identified in significant number of ears with hearing aids than ears without hearing aid (*P* = 0.003 and *P* = 0.006 respectively). Coagulase-negative staphylococci were the commonest identified bacteria. Others were Staphylococcus aureus, Pseudomonas aeruginosa, and Proteus species. Intolerable irritations of hearing aid wearing ears were significantly associated with bacterial and fungal otitis externa, and ear discharge (*P* = 0.005, 0.02, 0.03 respectively).

***Conclusions*** **:** This study demonstrates that using hearing aid alters the ear canal flora; increases risk of both fungal and bacterial otitis externa, as well as encourage wax debris formation, with resultant ear irritations. To ensure compliance their ears should periodically be attended to, by de-waxing or given topical antimicrobial agents where indicated.

## INTRODUCTION

Hearing aid (HA) users often present to otolaryngologist with complaints of constant irritations within the ear canals on account of either allergic contact dermatitis from the earmolds which connect the HA to the ear canal,^1^^,^^2^ or bacterial/fungal otitis externa,^3^^,^^4^ or wax impaction. The irritation, itching, and discharge from the ear canal usually present their challenges that sometimes make the use of HA difficult or even impossible. Some HA users get easily dissatisfied with the use of the aids regardless of the hearing benefits they derive. Some other users simply discontinue the use of the hearing aids on account of the irritations from debris accumulation in the canal. The most challenging scenario is with persistent bacteria/fungal otitis externa where the otolaryngologists sometimes consider fitting of the aids via more invasive alternative routes such as middle ear hearing implants.^5^^-^^7^ A wide range of contaminating bacteria and fungi growing on hearing aid surfaces as well as ear canals of HA users has been documented.^3^^,^^4^^,^^8^^,^^9^ However the growths of these microorganisms are yet to be related to the clinical state of canal with HA with and whether these organisms result in actual infection of the external auditory canals.

This study was carried out to examine the characteristics and microbial profile of ear canal debris, and to study the determinant factors of ear irritation in hearing aid users.

## METHODS

Thirty two hearing impaired individuals previously fitted with behind the ear HA, who arrived at the Ear nose and throat clinic of a tertiary health institution for routine hearing aid follow-up, or for HA related or some other complaints participated in this study.

Potential subjects were informed of the proposed study and asked to volunteer. Once subjects had given informed consent, their eligibility for inclusion was further evaluated. The approval of the study was given by the Institutional Ethical Review Committee.

These volunteers included individuals who were fitted with HAs in our department as well as those who were fitted elsewhere before presenting to our department. All the subjects were using the behind-the-ear HA. One was recruited if he/she has used a uni-aural HA for upwards of four weeks. Volunteers were excluded if they had active ear discharge originating from the middle ear in the presence of tympanic membrane perforation, or had history of penetrating trauma to the ipsilateral ear, or had taken any antimicrobial ear drops or systemic antibiotics in the preceding seven days.

On arrival at the clinic, potential subjects were interviewed and data concerning the age, duration of HA usage, history and frequency of ear irritation, pain, and discharge were enquired. The irritation was regarded as mild if occurs occasionally; moderate if it occurs almost all the time; severe if it is associated with ear pain, or discharge in the absence of any tympanic membrane perforation, or resulted in discontinuation of the use of HA. The ears were examined with otoscopes and the states of the ear canals and the ear drums recorded. Any debris/exudates seen were characterised. The ear canals were classified as either normal appearance; or as having otomycotic debris if it reveals fungal deposits/exudates; or bacterial exudates if there is purulent exudates; or wax debris; or a combination of the various debris.

Each volunteer had two ear swab samples taken, one each from the hearing aid-wearing ear canal and the other from the contralateral ear canal regardless of whether debris/exudates were found or not provided the tympanic membrane was intact. The samples from the contralateral ear served as the control. The samples were taken before cleaning the ear canals of any debris/exudates or cerumen, with sterile swab sticks which were enclosed in air tight plastic tubing and then transported to the microbiology test laboratory. The swabs were plated on MacConkey agar plates and incubated for 48 hours after which bacterial isolates and the antimicrobial sensitivities were identified using standard microbiological methods.^10^^,^^11^

***Statistical Analysis:*** The Statistical Package for Social Sciences (SPSS Inc, Chicago, IL, USA) version 16 was used for analysis. Chi square test was used to test the statistical significance of association between potential variables.

## RESULTS

A total of 64 ear swab samples, obtained from 32 hearing aid users, were analyzed. Seventeen male and 15 female patients (age range 9–78 years, mean 44.2 ± 17.2 years) were included in the study. There were complaints of irritations in 18 (56%) of the HA wearing ears and in 3 (9%) ears without HA. There were also complaints of pain (in 6/32 subjects, 19%) and discharge (in 4/32 subjects, 13%) in those ears in which a HA was worn.

Table-I outlines the nature of debris found in the HA wearing ears and the contralateral ears as well as their microbial isolates. Various forms of debris were identified in significant number of ears with HA (20 ears) compared to only 6 ears without HA (χ^2^ = 12.67; *p* = 0.003). Although ear wax accumulation seem slightly more identifiable in HA wearing ears compared to the contralateral ears without HA, the difference was not significant (χ^2^ = 0.988; *p* = 0.176).

The microbial cultures of the swabs from the HA wearing ears identified 9 bacteria and 7 fungal isolates compared to the significantly fewer isolates from ears without HA (χ^2^ = 8.60; *p* = 0.006). Regarding the details of the microbial isolates from the HA wearing ears, mixed bacterial and fungal isolates of *Proteus* and* Candida spps.* were identified in one of the ear swabs. Coagulase-negative staphylococci were isolated in five of the HA wearing ears and two ears without HA where otoscopy earlier revealed either no debris or wax. The rest of the cultures yielded other bacteria shown in Table-I were obtained from the HA wearing ears where otoscopy earlier identified obvious purulent exudates in the presence of an intact tympanic membrane.

Ear irritations were significantly documented more in the ears with HA (21), compared to three ears without HA (χ^2^ = 5.86; *p* = 0.021). In Table-II, the severity of ear irritations as indicated by the patient in the HA wearing ears were related to the otoscopy findings. It interestingly revealed that accumulation of canal debris were significantly associated with complaints of ear irritation (χ^2^ = 30.49; *p* = 0.002). Table-III shows the** a**nalysis of the association of different potential factors in relation to non-tolerance of the HA occasioned by severe ear irritations among the 32 HA users. Strong significant connections were found between severe intolerable ear irritations and presence of ear discharge, fungal ear infection, as well as bacteria otitis externa (*p* = 0. 03, *p* = 0.02, and *p* = 0.005 respectively).

## DISCUSSION

***Previously Reported Studies: ***The cul-de-sac characteristics of the external auditory canal encourages the growth of wide range of bacteria and fungi by virtue of its ability to trap moisture thereby predisposing the canal to the development of otitis externa.^3^^,^^10^ The occlusion of the canal by the ear moulds of the HA understandably increases the tendency for moisture retention within the canal thereby putting the HA users at a higher risk of otitis externa. It has been observed that when the canal is obstructed by a HA and its ear mould, it becomes an even warmer, darker and moister environment, which tends change the pH balance of cerumen in favour of alkaline pH and results in an environment conducive for microbial proliferation.^3^^,^^8^^,^^12^^,^^13^ In view of the aforementioned challenges, vented HA mould may present an advantage over the non-vented ones in creating room for ventilation of the ear canal and thereby reducing the chances of moisture retention within the canal. However, the vent in the ear mould may create room for an undesirable acoustic feed-back.

Paediatric or geriatric patients and those with immunocompromise have been identified to be at higher risk of developing localized or systemic infections due to even innocuous microorganisms.^3^^,^^4^^,^^8^^,^^9^

The proliferation of these microorganisms within the ear canal can potentially cause irritation of its lining and result in itching, otalgia, swelling, and ear discharge depending on the virulence and the immune status of the HA wearer.^9^ Beyond the irritation of the ear canals caused by the colonisation of the canals by microorganisms in HA users, the potentials for contact allergic reaction of the ear canal lining to the materials of the ear moulds have been identified as significant cause of ear irritation in HA wearers.^1^^,^^2^

***The present study in comparison with other studies: ***The data reported in this study indicate the presence of both bacterial and fungal microbial growth in the canals of both the ears with HA and without. There was significant difference between the microbial growths recovered from HA wearing ears and the contralateral ears without HA similar to the reports in a study that reported significant differences between hearing aid-wearing ears and ears without hearing aids regarding the microbial flora.^3^ Although Mehdinejab et al.^9^ found no significant difference between children wearing HA and the children without HA regarding the ear canal bacterial flora, they however documented more frequent bacteria isolates from the ears with HA (59%) than ears without HA (41%).

Coagulase-negative staphylococci are usually considered to be non-pathogenic and their role in otitis externa is still largely unknown. They have been cultured in the normal external auditory canal, either alone or in combination with other organisms, including diphtheroids or occasional fungal spores^8^^,^^13^ In this present study they were cultured in ears that showed no clinical evidence of otitis externa, but more in ears with HA than those without HA. This seems to suggest that HA encourages the proliferation of not only pathogenic bacteria causing otitis externa, but also non-pathogenic bacteria with potential for producing ear irritation.

Ear irritations were documented in significant numbers of the ears with HA compared to contralateral ears without HA in this study. Furthermore, in the ears with HA, 89% of these irritations were noted in ears where debris were identified in the canal, including wax as was outlined in Table-II. The data in this study shows that both wax and infective debris accumulations were clearly more prevalent in HA wearing ears than in contralateral ears without HA (*P* = 0.003). It is therefore conceivable that hearing aid mould increases the tendency for ear canal irritations by encouraging proliferation of both pathogenic and non-pathogenic microorganisms, as well as accumulation of wax debris. However, the ear irritations were more severe in the presence of bacteria and/or fungal infections than in ears with only wax debris.

Although fungal otitis externa was significantly associated with intolerable irritations in this study, the data indicated that bacteria otitis externa constitute the most significant factor for discontinuation of HA usage due to resulting severe intolerable ear irritation. Bacterial infections are obviously likely to be associated with inflammation of the ear canal, and are understandably more likely to result in ear pain as well as discharge, which will make the continued use of HA difficult or perhaps impossible. Users that experience both ear pain and discharge resulting from bacteria otitis externa will most likely discontinue the HA pending their resolutions.

Some of the subjects in this study justifiably blamed their HA for the irritations and ordeal they underwent, and often contemplated the option of abandonment of the HA. It is therefore instructive for periodic attention to be paid to the care of their ears, by de-waxing or given topical antimicrobial agents where indicated, as part of measures to improve the comfort and ensure compliance of HA usage. Some authors have recommended education of HA users in the use of an appropriate hygiene routine to clean and disinfect hearing aids and ear moulds in order to avoid otitis externa, by wiping the surfaces with a tissue soaked in a non-alcohol-based disinfectant which should be stored in an appropriate case for later use.^3^^,^^8^^,^^9^ It has been demonstrated that Cleaning with 70 per cent alcohol solution was ineffective, in particular for pathogenic fungi on ear moulds.^4^

It is of interest to observe that not all the ear irritations documented in this study resulted from wax or infective debris as two of the patients reported irritations in their HA wearing ears in absence of any documented debris in the canal on otoscopy. The irritation was even so severe to the extent that the use of HA was discontinued in one of them. The patient was only able to recommence the use of the HA after the resolution of the troublesome irritation following use of oral doses of antihistamine and steroid-based ear drop. It seems reasonable to believe that the irritations in these two cases were occasioned by contact allergic reaction to the HA ear moulds, or due to mechanical pressure exerted on the lining of the canal by the ear mould. Ear irritations due to contact allergic reactions to HA ear moulds have been reported.^1^^,^^2^ In one particular series, contact allergy to the ear mould material was found in 27% of the patients, where a positive test reaction were documented to methyl methacrylate, triethyleneglycol dimethacrylate and urethane di-methacrylate skin patches.^1^

**Table-I:** Ear Debris and Microbial Culture Analysis in Hearing Aid wearing Ears and Ears without Hearing Aids

*Aids*	*HA Wearing Ears*	*Ears without Hearing*	*p Value*
*Ear Debris*			
No debris	12	26	0.003
Wax	9	5	0.176
Bacterial exudates	4	0	0.048
Fungal deposits	6	1	0.036
Mixed Fungal/bacterial exudates	1	0	-
*Culture Result Analysis*			
No Growth	17	29	0.006
*Pseudomonas aeruginosa*	1	0	
*Staphylococci aureus*	2	0	
Coagulase-negative Staphylococci	5	2	
*Proteus spp.*	1	0	
*Candida* spp.	7	1	

**Table-II:** Ear Irritations in Relation to the Nature of Canal Debris in the Ears with Hearing Aids. N = 32

*Ear Debris*					
	*No debris*	*Wax debris*	*Bacterial exudates*	*Fungal debris*	*Mixed* *
*Ear Irritation *					
Nil	10	4	0	0	0
Mild	1	3	0	3	0
Moderate	0	2	1	1	1
Severe (Intolerable)	1	0	3	2	0

* *Combined bacterial and fungal exudates*

**Table-III:** Determinant Factors for Non-tolerance of the Hearing Aid

*Factors*	*F or Chi value*	*p Value *
Age	0.14	0.71
Duration of HA usage	0.27	0.60
Associated Otalgia	0.93	0.34
Ear discharge	5.61	0.03
Wax in the Ear	0.94	0.36
Fungal otitis externa	6.01	0.02
Bacteria otitis externa	9.27	0.005

## CONCLUSION

In conclusion, wearing HA seems to modify the microbial flora of the ear canal. It encourages proliferation of not only the commonest non pathogenic coagulase negative Staphylococci, but also the pathogenic bacteria and thereby increases the risk of otitis externa. Although the resulting ear irritations seem to be most severe in the presence of bacteria as well as fungal otitis externa, it is also significantly observed with wax debris in the canal. Finally, the possibility of contact allergic dermatitis developing due to sensitization of the ear canal to ear mould material should be considered in HA users presenting with severe ear irritation in the absence of obvious ear canal debris.

## Notes

***Source of Funding: ***Self.
